# The State of Patient Engagement among Pain Research Trainees in Canada: Results of a National Web-Based Survey

**DOI:** 10.1080/24740527.2022.2115879

**Published:** 2022-10-19

**Authors:** Kyle Vader, Perri R. Tutelman, Delane Linkiewich, Catherine Paré, Alice Wagenaar-Tison, Kathryn A. Birnie, Christine T. Chambers, Kathleen Eubanks, Nader Ghasemlou, Janet Gunderson, Maria Hudspith, Therese Lane, Jordan Miller, Dawn P. Richards

**Affiliations:** a School of Rehabilitation Therapy, Queen’s University, Kingston, Ontario, Canada; b Department of Psychology & Neuroscience, Dalhousie University, Halifax, Nova Scotia, Canada; c Chronic Pain Network, McMaster University, Hamilton, Ontario, Canada; d Department of Psychology, University of Guelph, Guelph, Ontario, Canada; e Department of Psychology, McGill University, Montréal, Québec, Canada; f Department of Anatomy, Université du Québec à Trois-Rivières, Trois-Rivières, Québec, Canada; g Department of Anesthesiology, Perioperative and Pain Medicine, University of Calgary, Calgary, Alberta, Canada; h Department of Pediatrics, Dalhousie University, Halifax, Nova Scotia, Canada; i Departments of Anesthesiology & Perioperative Medicine and Biomedical & Molecular Sciences, Queen’s University, Kingston, Ontario, Canada; j Pain BC, Vancouver, British Columbia, Canada; k Five02 Labs Inc, Toronto, Ontario, Canada

**Keywords:** Pain, survey, trainees, patient engagement, Patient-Oriented Research

## Abstract

**Background:**

Patient engagement (PE) in research refers to partnering with people with lived experience (e.g., patients, caregivers, family) as collaborators in the research process. Although PE is increasingly being recognized as an important aspect of health research, the current state of PE among pain research trainees in Canada is unclear.

**Aims:**

The aims of this study were to describe perspectives about and experiences with PE among trainees conducting pain research in Canada, to identify perceived barriers and facilitators, and to describe recommendations to improve its implementation.

**Methods:**

A cross-sectional web-based survey (English and French) was administered to trainees at any level conducting pain research at any Canadian academic institution.

**Results:**

A total of 128 responses were received; 115 responses were complete and included in the final analysis. The majority of respondents identified as women (90/115; 78.3%), in graduate school (83/115; 72.2%), and conducting clinical pain research (83/115; 72.2%). Most respondents (103/115; 89.6%) indicated that PE is “very” or “extremely” important. Despite this, only a minority of respondents (23/111; 20.7%) indicated that they “often” or “always” implement PE within their own research. The most common barrier identified was lack of knowledge regarding the practical implementation of PE, and understanding its positive value was the most commonly reported facilitator. Recommendations for improving the implementation of PE were diverse.

**Conclusions:**

Despite viewing PE as important in research, a minority of pain research trainees regularly implement PE. Results highlight perceived barriers and facilitators to PE and provide insight to inform the development of future training and other enabling initiatives.

## RÉSUMÉ

**Contexte**: L'engagement des patients dans la recherche fait référence au partenariat avec des personnes ayant une expérience vécue (p. ex. des patients, des soignants ou des membres de la famille) en tant que collaborateurs dans le processus de recherche. Bien que l'engagement des patients soit de plus en plus reconnu comme un aspect important de la recherche en santé, son état actuel chez les stagiaires en recherche sur la douleur au Canada n’est pas clair.

**Objectif**: Les objectifs de cette étude étaient de décrire les points de vue et les expériences sur l'engagement des patients chez les stagiaires menant des études sur la douleur au Canada, de recenser les obstacles et les facilitateurs perçus et de formuler des recommandations pour améliorer sa mise en œuvre.

**Méthodes**: Une enquête transversale sur le Web (en anglais et en français) a été menée auprès des stagiaires de tout niveau menant des études sur la douleur dans n'importe quel établissement universitaire canadien.

**Résultats**: Au total, 128 réponses ont été reçues; 115 réponses étaient complètes et ont été incluses dans l'analyse finale. La majorité des répondants ont indiqué qu'elles étaient des femmes (90/115; 78,3 %), qu'elles étaient inscrites aux cycles supérieures (83/115 ; 72,2 %) et qu'elles effectuaient des études cliniques sur la douleur (83/115 ; 72,2 %). La plupart des répondants (103/115 ; 89,6 %) ont indiqué que l'engagement était « très » ou « extrêmement » important. Malgré cela, seule une minorité de répondants (23/111; 20,7 %) ont indiqué qu'ils mettaient en œuvre l'engagement des patients « souvent » ou « toujours » dans leur propre recherche. L'obstacle le plus fréquemment énoncé était le manque de connaissances concernant la mise en œuvre pratique de l'engagement des patients, et la compréhension de sa valeur positive était le facilitateur le plus souvent signalé. Les recommandations visant à améliorer la mise en œuvre de l'engagement des patients étaient diverses.

**Conclusions**: Bien que l'engagement des patients soit considéré comme important dans la recherche, une minorité de stagiaires en recherche sur la douleur le mettent régulièrement en œuvre. Les résultats mettent en évidence les obstacles et les facilitateurs perçus pour l'engagement des patients et fournissent un aperçu pour éclairer l'élaboration de la formation future et d'autres initiatives habilitantes.

## Introduction

The Canadian Institutes of Health Research defines patient engagement (PE) in research as “meaningful and active collaboration [of patients] in governance, priority setting, conducting research, and knowledge translation.”^1^ Within this definition, the term “patient” is defined as an “overarching term inclusive of individuals with personal experience of a health issue and informal caregivers, including family and friends.”^1^ PE is increasingly being recognized as an important component of the health research process to facilitate patient-centered research priorities and improve patient outcomes at both the individual and community level.^1^

Existing frameworks, such as the Strategy for Patient-Oriented Research–Patient Engagement Framework by the Canadian Institutes of Health Research, highlight the importance of inclusiveness, support, mutual respect, and co-building as guiding principles of PE.^1^ A growing body of literature suggests that successful PE requires authentic partnership,^2,3^ effective communication,^4^ engagement throughout the research process,^5^ and adequate training for both researchers and patient partners.^6^ Given the potential benefits (e.g., increased impact of study findings),^7–9^ challenges (e.g., tokenism),^10–12^ and practical considerations (e.g., authorship on publications and outputs)^13^ of PE, it is important to understand experiences with PE from all stakeholders within the research enterprise, including patient partners, researchers, and research trainees.

In recent years, there has been increasing recognition of the value of PE within the field of pain in Canada (e.g., creation of the Chronic Pain Network,^14^ Solutions for Kids in Pain,^15^ and Chronic Pain Center of Excellence for Canadian Veterans,^16^ which all include PE as a central pillar; engagement of patients within the Canadian Pain Society^17^ and Canadian Pain Task Force^18^). Despite this, the current state of PE among pain research trainees (i.e., undergraduate students, health professions students, graduate students, postdoctoral fellows, residents) has not yet been described in the literature. Describing the current state of PE among pain research trainees, the pain research leaders of tomorrow, will provide foundational knowledge that can be used to better support trainees to carry out PE within their own research.

The purpose of this study was to describe perspectives about and experiences with PE among trainees conducting pain research in Canada, to identify perceived barriers and facilitators, and to describe recommendations to improve its implementation.

## Materials and Methods

### Research Team

Our research team included patient partners (DL, KE, JG, TL); pain research trainees (KV, PRT, CP, AWT); biomedical, clinical, and translational science researchers (KAB, CTC, NG, JM); and individuals who facilitate PE in research (MH, DPR). The individuals on this team were purposely selected to ensure that the relevant stakeholders were engaged. Patient partners played a critical role throughout this research: helping construct study objectives, developing the web-based survey, assisting with recruitment/survey distribution, contributing to data analysis, providing feedback on and involvement in the writing of this article, and participating in knowledge mobilization activities.

### Study Design

We implemented a cross-sectional, web-based survey in English and French.^19^ Ethics approval was granted by the Queen’s University Health Sciences and Affiliated Teaching Hospitals Research Ethics Board in Kingston, Ontario, Canada (HSREB No. 6030467). All participants provided informed consent to participate in this study via their response to the first question of the web-based survey.

### Survey Development

We developed a web-based survey based on our study objectives in Qualtrics, Provo, UT. We chose to include a combination of open- and closed-ended questions to enhance ease of survey completion. Two members of the research team (KV, DPR) led the survey creation. Closed-ended survey response options (e.g., questions related to perceived barriers and facilitators) were constructed based on the research team’s own lived experiences implementing PE. Other research team members, including patient partners, reviewed the survey and provided suggestions that were incorporated to improve the content, structure, and clarity of questions.

After we created an English version, we translated our survey to French. Two bilingual members of the research team (CP, AWT) led the survey translation. In alignment with best practices, we first translated our survey from English to French, followed by a translation back to English to ensure consistency with the original English version.^20^

Before launching our survey, three members of the research team (KV, DL, AWT) conducted cognitive interviews of our survey with two pain research trainees (one with the English version and one with the French version). Cognitive interviewing helps to improve survey design by having an informed individual (i.e., a pain research trainee) think out loud while completing a survey in front of the designer.^21^ As a result of the cognitive interviews, we revised some of the questions to improve clarity and/or flow. The final survey included 20 questions. See Supplemental Files 1 and 2 for English and French versions of the survey, respectively.

### Sample

We used a convenience sampling technique to recruit participants to complete our survey.^22^ Individuals could participate in our research if they were (1) able to complete a web-based survey in English or French, (2) a current trainee (i.e., undergraduate student, health professions student, graduate student, postdoctoral fellow, or resident) at a Canadian academic institution, and (3) engaged in research on a topic related to the field of pain.

### Recruitment

Potential participants were invited to complete our survey using a multipronged recruitment approach, including e-mails through relevant pain research Listservs as well as social media posts from pain-related organizations in Canada (e.g., Chronic Pain Network, PainBC). Individual members of the research team also posted recruitment flyers to their personal social media accounts. Participants were invited to complete the survey from September to November 2020.

### Data Analysis

We analyzed responses using descriptive statistics (19 closed-ended questions) and content analytic techniques (one open-ended question).^13^ Two members of the research team (KV, DPR) led the qualitative content analysis of responses to the open-ended question.^23^ The inductive content analysis followed procedures described by Elo and Kyngäs, including preparation, organizing, and reporting of the data.^23^

## Results

We received a total of 128 survey responses. Thirteen survey responses were excluded because respondents did not complete our survey beyond the demographic questions. We included the completed 115 survey responses (105 English and 10 French) in our final analysis.

### Respondents’ Characteristics

Respondents (see Table 1) had a median age of 27 years (interquartile range, 24, 32). The majority of respondents identified as follows: women (90/115; 78.3%), being in graduate school as a master’s student or a PhD student/candidate (83/115; 72.2%), and conducting clinical pain research (83/115; 72.2%). Respondents reported attending academic institutions across Canada, with the majority studying at institutions in Ontario (60/115; 52.2%) and Quebec (24/115; 20.9%).

**Table 1.** Respondent characteristics.

Characteristic	Details (%)
Age in years, median (interquartile range) (*n* = 115)	27 (24, 32)
Gender, *n* (%) (*n* = 115)	
Woman	90 (78.3)
Man	22 (19.1)
Gender-fluid, nonbinary, and/or Two-Spirit	2 (1.7)
Did not identify with options provided	1 (0.9)
Province/territory of academic institution, *n* (%) (*n* = 115)	
Alberta	4 (3.5)
British Columbia	2 (1.7)
Manitoba	1 (0.9)
New Brunswick	0 (0.0)
Newfoundland and Labrador	1 (0.9)
Northwest Territories	0 (0.0)
Nova Scotia	7 (6.1)
Nunavut	0 (0.0)
Ontario	60 (52.2)
Prince Edward Island	0 (0.0)
Quebec	24 (20.9)
Saskatchewan	5 (4.3)
Yukon	0 (0.0)
Status as a trainee, *n* (%) (*n* = 115)	
Undergraduate student, non-health profession	15 (13.0)
Undergraduate student, health profession	3 (2.6)
Master’s student, non-health profession	12 (10.4)
Master’s student, health profession	11 (9.6)
Combined health profession program and Master’s student	2 (1.7)
PhD student/candidate, non-health profession	26 (22.6)
PhD student/candidate, health profession	31 (27.0)
Combined health professions and PhD student/candidate	1 (0.9)
Postdoctoral fellow	11 (9.6)
Other	2 (1.7)
Area of pain research, *n* (%)* (*n* = 115)	
Clinical science (e.g., pain research that focuses on individuals with pain, such as lived experience, assessment, interventions, or measurement)	83 (72.2)
Basic science (e.g., pain research that focuses on cells, proteins, molecules)	52 (45.2)
Translational–basic to clinical science (e.g., pain research that bridges the gap between basic and clinical research)	17 (14.8)
Translational–clinical science to broader community (e.g., pain research that bridges the gap between clinical research and the general public/health care providers/policymakers)	12 (10.4)
Mechanism of funding to support trainee salary, *n* (%)* (*n* = 115)	
Noncompetitive internal funding package from academic institution	39 (33.9)
Provincial graduate scholarship	30 (26.1)
Tri-Council Canada Graduate Scholarship (i.e., Social Sciences and Humanities Research Council, Natural Sciences and Engineering Research Council, Canadian institutes of Health Research)	29 (25.2)
No funding	25 (21.7)
Competitive internal academic institution award	14 (12.2)
Charity/not-for-profit scholarship	6 (5.2)
Other	11 (9.6)
Own experience with pain (personal or family member) motivated to pursue pain research, *n* (%) (*n* = 115)	
Yes	57 (49.6)
No	58 (50.4)
Career stage of primary research supervisor, *n* (%) (*n* = 115)	
Early career (i.e., full-time, independent research appointment for 0–5 years)	28 (24.3)
Mid-career (i.e., full-time, independent research appointment for 5–15 years)	42 (36.5)
Senior career (i.e., full-time, independent research appointment for >15 years)	35 (30.4)
Not sure	9 (7.8)
Primary research supervisor implements PE in research, *n* (%) (*n* = 112)	
Yes	67 (59.8)
No	23 (20.5)
Not sure	22 (19.6)

*Survey respondents could endorse more than one response option.

### Perspectives about and Experiences with PE in Research

Respondents reported perspectives about and experiences with PE in research (see Table 2). The overwhelming majority of respondents (103/115; 89.6%) indicated that PE in pain research is “very” or “extremely” important. Although most respondents acknowledged the importance of PE in pain research, a minority of respondents (23/111; 20.7%) indicated that they “often” or “always” implement PE within their own research as the primary researcher. The most common ways in which respondents described implementing PE in pain research as the primary researcher included involving a patient to advise on or assist with participant recruitment or with materials for participants (48/107; 44.6%), involving a patient in the planning stage of research (25/107; 23.4%), and involving a patient in data collection or analysis (18/107; 16.8%).

**Table 2.** Perspectives about and experiences with PE in research.

Characteristic	No. (%) of respondents
Importance of PE in pain research (*n* = 115) Not at all Slightly Moderately Very Extremely	3 (2.6) 0 (0.0) 9 (7.8) 48 (41.7) 55 (47.8)
Training received on PE in research* (*n* = 115) Formal training as part of academic program (e.g., as part of an academic course) Formal training outside of academic program (e.g., a conference workshop or webinar) Informal training as part of academic program (e.g., mentorship from a peer/supervisor as part of academic program) Informal training outside of academic program (e.g., mentorship from a peer/non-supervisor outside of academic program) No training received	65 (56.5) 42 (36.5) 42 (36.5) 26 (22.6) 28 (24.3)
Knowledge about PE in research (*n* = 111) Not at all Slightly Moderately Very Extremely	9 (8.1) 35 (31.5) 51 (45.9) 13 (11.7) 3 (2.7)
Confidence in ability to implement PE in research (*n* = 111) Not at all Slightly Moderately Very Extremely	13 (11.7) 30 (27.0) 48 (43.2) 18 (16.2) 2 (1.8)
Frequency implementing PE in research on projects as the primary researcher (*n* = 111) Never Rarely Sometimes Often Always	48 (43.2) 17 (15.3) 23 (20.7) 14 (12.6) 9 (8.1)
Frequency implementing PE in research on projects when not the primary researcher (*n* = 110) Never Rarely Sometimes Often Always	46 (41.8) 21 (19.1) 28 (25.5) 13 (11.8) 2 (1.8)
Implementation of PE in research on projects when the primary researcher* (*n* = 107) Involving a patient to advise or assist with participant recruitment or with materials for participants Presenting ideas to patients for input/feedback Involving a patient in the planning stage of research Involving a patient in data collection or analysis Involving a patient in knowledge translation Involving a patient on a consultant basis Involving a patient in manuscript writing Involving a patient in grant writing Other (i.e., using own lived experience to inform research broadly) Have not implemented PE in research on a project when the primary researcher	48 (44.6) 31 (34.6) 25 (23.4) 18 (16.8) 17 (15.9) 17 (15.9) 9 (8.4) 5 (4.7) 2 (1.8) 54 (50.5)
Implementation of PE in research on projects when not the primary researcher* (*n* = 106) Involving a patient to advise or assist with participant recruitment or with materials for participants Presenting ideas to patients for input/feedback Involving a patient in the planning stage of research Involving a patient in data collection or analysis Involving a patient on a consultant basis Involving a patient in knowledge translation Involving a patient in manuscript writing Involving a patient in grant writing Other (i.e., serving as a patient partner based on own lived experience) Have not implemented PE in research on a project when not the primary researcher	34 (32.1) 28 (26.4) 24 (22.6) 20 (18.9) 19 (17.9) 16 (15.1) 13 (12.3) 9 (8.5) 1 (0.9) 51 (48.1)

*Survey respondents could endorse more than one response option.

### Perceived Barriers and Facilitators to Implementation of PE in Research

Barriers and facilitators to PE were also identified by respondents (see Table 3). The three most highly endorsed barriers included being unsure how to practically implement PE (41/105; 39.0%), a lack of funding to reimburse a patient partner or provide them with compensation for participation (36/105; 34.3%), and not knowing how to find patient partners (29/105; 27.6%). Respondents’ three most endorsed facilitators to implementing PE in research as a pain research trainee included seeing the value of PE (48/108; 44.4%), having support from their supervisor (44/108; 40.1%), and knowledge of how to practically implement PE (26/108: 24.1%).

**Table 3.** Perceived barriers and facilitators to implementation of PE in research.

Characteristic	No. (%) of respondents
Barriers when implementing PE in research* (*n* = 105) Unsure how to practically implement PE Lack of funding to reimburse a patient partner or provide them with compensation for participation Don’t know how to find patient partners Lack of confidence to implement PE Lack of support from department Lack of support from institution Lack of support from supervisor Unsure about the value of PE Other (e.g., impacts of COVID-19 pandemic, perception that PE is not relevant to type/topic of research, practical issues related to research ethics) No perceived barriers	41 (39.0) 36 (34.3) 29 (27.6) 28 (26.7) 12 (11.4) 10 (9.5) 9 (8.6) 6 (5.7) 11 (10.5) 18 (17.1)
Facilitators when implementing PE in research* (*n* = 108) See the value of PE Support from supervisor Knowledge of how to practically implement PE Ability to find patient partners Availability of funding to reimburse a patient partner or provide them with compensation for participation Confidence to implement patient PE Support from institution Support from department No perceived facilitators Other Not applicable—had not implemented PE in research	48 (44.4) 44 (40.1) 26 (24.1) 25 (23.1) 24 (22.2) 19 (17.6) 14 (13.0) 11 (10.2) 7 (6.5) 0 (0) 30 (27.8)

*Survey respondents could endorse more than one response option.

### Recommendations to Improve the Implementation of PE in Research

Respondents (*n* = 63) provided recommendations to improve the implementation of PE among pain research trainees. From our inductive content analysis, we constructed four categories to describe respondents’ recommendations, including (1) improve availability and accessibility of training opportunities and resources on PE in research, (2) provide more funding opportunities that support and/or require PE in research, (3) create systems to support trainees to find patient partners, and (4) ensure that supervisors, departments, and institutions support and encourage PE in research. See Table 4 for categories, category descriptions, and representative quotations.

**Table 4.** Categories, category descriptions, and supporting quotations related to recommendations to improve the implementation of PE in research.

Category	Category description	Supporting quotations
Improve availability and accessibility of training opportunities and resources on PE in research	Respondents described a need for improved availability and accessibility of training opportunities on PE in research for trainees both within and outside of formal academic programs. Many respondents described a desire for training to focus on hands-on and practical learning. Respondents expressed a desire for easy-to-follow resources on how to effectively conduct PE as a trainee. Some respondents also acknowledged that many resources do already exist but could be better combined and found in one accessible virtual location.	“More formal training available, including step-by-step examples of how to engage patients.” “Formal education on practical aspects of how to engage patients in research would be beneficial at the beginning of an academic experience (e.g., start of graduate program), so that trainees can build this into their proposal from the onset.” “Develop webinars freely available to all [trainees and patients] about patient engagement in pain research. Live events online could promote networking between trainees and patients interested in patient engagement in pain research.” “Lots of resources are available (papers, webinars, patient partner groups, etc.); some trainees might not be aware of these resources. So a collection of these resources in an accessible/visible place may help.”
Provide more funding opportunities that support and/or require PE in research	Respondents described a desire for improving funding opportunities to support and/or require PE in research. Respondents expressed a desire for improving opportunities for funding agencies and institutions/departments to support compensation for patient partners’ contributions in research. Some respondents also emphasized that funding agencies should prioritize PE as a requirement among trainee scholarship applications (e.g., Tri-Council agencies).	“Provide funding for trainees to provide honorariums for patient[s] engaged in their research.” “Make [patient engagement] a mandatory component of provincial or federal funding.” “I think there should be opportunities for trainees to seek out funding to support having a patient involved in their research (e.g., from [the Canadian Institutes of Health Research] or within specific departments or graduate schools broadly).” “Funders need to appreciate the role of patients in the field of pain research and encourage trainees to include patients in research.”
Create systems to support trainees to find patient partners	Respondents described a recommendation to create systems to support trainees to find patient partners. Several respondents described the potential benefit of a registry or matching system to connect pain research trainees (and pain researchers more broadly) with patient partners who have lived experience of certain pain conditions to facilitate the process of PE in research.	“It would be helpful to have a platform where researchers and patients can be matched based on interest and pain diagnosis.” “Have a list of places where to get patient partners for trainee research, specifically for Canada and specifically for pain (perhaps even a registry).” “Having a better system to match trainees with interested patient partners. It’s particularly challenging when you are a clinical trainee because of the ethics of approaching/partnering with patients in settings where you also provide care.” “A registry of patients who are willing to engage, and their area/topics of interest would be helpful.”
Ensure that supervisors, departments, and institutions support and encourage PE in research	Respondents reported a need for supervisors, departments, and institutions to support and encourage PE in research. Many respondents described that if their supervisors and departments/institutions themselves implemented and recognized the value of PE, this would make it easier for pain research trainees to implement PE in their research.	“Have the institution and departments explicitly encourage their faculty to implement patient engagement in their research and involve their trainees in this experience.” “Provide more education to supervisors in all types of research (e.g., basic science) in how to involve PE in their research/student’s research and also how to support students who want to implement PE in their research.” “More education is need[ed] at the institutional level to prioritize patient engagement.” “If patient engagement and patient-led research is made to be the standard among supervisors, trainees will inevitably learn and engage in those practices.”

## Discussion

To our knowledge, this is the first study to survey pain research trainees across Canada to describe their perspectives about and experiences with PE, to identify perceived barriers and facilitators, and to describe recommendations to improve its implementation. The results provide foundational knowledge on the state of PE among pain research trainees in Canada and have potential implications to inform future training opportunities and other enabling initiatives to support the successful implementation of PE among this group of up-and-coming researchers. Though the focus of this work was on trainees within the pain research community, findings may be transferable to trainees in other research disciplines.

The overwhelming majority of respondents reported that PE is “very” or “extremely” important in pain research, suggesting that pain research trainees in Canada perceive the value of PE. Increasing recognition of the importance of PE in health research, and specifically within the field of pain in Canada,^14–18^ may explain why respondents had overall positive views toward PE. Furthermore, given the fact that most respondents indicated that they conducted clinical pain research, it is possible that this cohort of pain research trainees is more familiar with PE than those conducting other types of pain research, such as basic science. Although respondents had positive views, only a small minority indicated that they “often” or “always” implement PE in projects where they are the primary researcher (e.g., their thesis or dissertation research). This is an important finding and suggests that there are opportunities to move beyond raising awareness and support the actual implementation of PE among pain research trainees. Furthermore, we found that a minority of respondents indicated feeling “very” or “extremely” knowledgeable or confident about implementing PE in research, despite the majority indicating that they had received formal training on the topic as part of their academic program. This indicates that there are opportunities to improve the quality and depth of training provided in this context. Our results suggest that training opportunities, such as those within and outside of academic programs, should focus on theoretical and practical knowledge acquisition and confidence building to support successful implementation of PE in research. Future work, such as initiatives conducted by the Strategy for Patient-Oriented Research National Training Entity,^24^ a national platform supported by the Canadian Institutes of Health Research with the goal of advancing patient-oriented research, could also explore the development of competencies and best practices for training on PE that are specifically tailored to meet the needs of research trainees, including those conducting pain research.

The most commonly endorsed barriers when implementing PE in research among respondents were being unsure how to practically implement PE, lack of funding to reimburse/compensate patient partners, and not knowing how to find patient partners. The most common facilitators endorsed by respondents included seeing the value of PE, support from their supervisor, and knowledge of how to practically implement PE. Our findings align with previous research by Heckert and colleagues that explored perceived challenges to the successful implementation of PE, including the importance of infrastructure and supports, such as funding support to appropriately compensate patient partners for their expertise and contributions.^25^ Because the most common barrier to implementing PE among our respondents was being unsure how to practically implement PE, this suggests that training and mentorship opportunities should specifically focus on practical and real-world application. Online resources do already exist on how to practically implement PE in research, such as resources created by the Chronic Pain Network^26^ and provincial Strategy for Patient-Oriented Research Support Units across Canada (e.g., Ontario Strategy for Patient-Oriented Research Support Unit).^27^ As such, it may be important to focus efforts on more widely advertising and disseminating these resources. However, given the limitations of passive knowledge translation strategies (e.g., readings and didactic education),^28^ innovative and engaging training models (e.g., mentorship programs between trainees and PE experts) could also be developed and evaluated as a strategy to improve trainees’ confidence in practically carrying out PE. Finally, because respondents reported that supervisors are an important facilitator of PE, future efforts should ensure that researchers are equipped with the knowledge, skills, and confidence to appropriately support pain research trainees to carry out PE.

Respondents provided diverse recommendations to improve the implementation of PE among trainees conducting pain research in Canada. Recommendations related to improving the availability and accessibility of training, creating more funding opportunities to compensate patient partners, implementing systems to support trainees to find patient partners, and ensuring that supervisors, departments, and institutions provide support and encouragement. These recommendations have important implications to improve the implementation of PE among pain research trainees. For example, the importance of appropriate remuneration for patient partners’ skills, time, and expertise as research team members has been widely recognized.^29^ To combat compensation barriers, initiatives could be made by pain research specific organizations (e.g., Canadian Pain Society) and other research funders (e.g., Canadian Institutes of Health Research) to create targeted funding competitions that provide trainees with the necessary funds to implement PE within their own research. Additionally, because respondents recommended creating systems to link pain research trainees with patient partners, this highlights an opportunity for relevant organizations and institutions to create initiatives and/or programs to facilitate connections between trainees and patient partners. Given the practical implications of these findings, the recommendations described by survey respondents were used to inform the proposed training and mentoring initiatives within the application to the Canadian Institutes of Health Research for continued funding of the Chronic Pain Network,^14^ which will focus on knowledge mobilization and implementation science.

### Limitations

This research has potential limitations that need to be considered when interpreting the results. First, because this was a self-report web-based survey, there may be important differences between what respondents reported and what happens within their day-to-day research practice related to PE due to response bias. Second, given our respondent demographics (e.g., majority women conducting clinical pain research), our findings may not be as representative of men and gender-fluid pain research trainees as well as those conducting nonclinical pain research (e.g., basic science). Third, it is possible that those who responded to our survey were more familiar or interested in PE than the typical pain research trainee due to participation bias. Finally, because we limited our survey to pain research trainees across Canada, it is unclear how our findings translate to research trainees in other fields and countries. Given variations in PE norms and practices across fields and geographic jurisdictions, this may be a valuable area of exploration for future research.

## Conclusions

Most pain research trainees in Canada perceive that PE in research is important. Despite this, a minority regularly implement PE within their own research. The most common barrier identified was lack of knowledge regarding the practical implementation of PE, and understanding its positive value was the most commonly reported facilitator. Recommendations to improve the implementation of PE related to training and resources, funding/compensation for patient partners, creating initiatives that facilitate connections with patient partners, and the importance of support from supervisors, departments, and institutions. These results provide a foundation to understand the current state of PE among pain research trainees in Canada and have implications to inform the development of training opportunities and other enabling initiatives to support the successful implementation of PE among this group of up-and-coming researchers.

## Supplementary Material

Supplemental Material

Supplemental Material
