# Supplementary material for: The State of Patient Engagement among Pain Research Trainees in Canada: Results of a National Web-Based Survey
Source: Can J Pain. 2022 Oct 19;6(1):185–94. doi: 10.1080/24740527.2022.2115879 (PMC9586693; doi:10.1080/24740527.2022.2115879)
Supplement: Supplemental Material [file UCJP_A_2115879_SM1847.pdf]

Supplemental File 2 – Survey (French)

Q1: Quel est votre âge (en années)? [ouvrir la zone de texte]

Q2: A quel genre vous identifiez-vous?
- Femme
- Homme
- Genre fluide, non-binaire, et/ou bispirituel.le
- Je ne m'identifie à aucunes options mentionnées ci-haut (Veuillez décrire comment vous vous identifiez) : [ouvrir la zone de texte]

Q3: Dans quelle province se trouve votre institution académique actuelle?
- Alberta
- Colombie Britannique
- Manitoba
- Nouveau Brunswick
- Terre Neuve et Labrador
- Territoires du Nord-Ouest
- Nouvelle Écosse
- Nunavut
- Ontario
- Île du Prince-Édouard
- Québec
- Saskatchewan
- Yukon

Q4: Quelle catégorie décrit le mieux votre statut en tant qu'étudiant-chercheur?
- Étudiant de premier cycle, hors professions de la santé
- Étudiant de premier cycle, professions de la santé (p. e. IA, MD)
- Étudiant à la maîtrise, hors professions de la santé
- Étudiant à la maîtrise, professions de la santé (p. e. PT, ET)
- Combinaison de programme de profession de la santé et étudiant à la maîtrise (p. e. MD/MSc)
- Étudiant/candidat au doctorat, hors professions de la santé
- Stagiaire postdoctoral.e, hors professions de la santé
- Stagiaire postdoctoral.e, professions de la santé (p. e. psychologie clinique)
- Combinaison de programme de profession de la santé et étudiant/candidat au doctorat (p. e. MD/PhD)
- Résident en médecine (p. e., médecin résident)
- Autre (veuillez décrire) : [ouvrir la zone de texte]

Q5: À quel stade de sa carrière en est votre directeur de recherche principal?

- Début de carrière (p.e. plein temps, nomination de chercheur indépendant pour 0-5 ans)
- Milieu de carrière (p.e. plein temps, nomination de chercheur indépendant 5-15 ans)
- Carrière senior (p.e. plein temps, nomination de chercheur indépendant > 15 ans)
- Je ne suis pas certain.e.

Q6: Quelle(s) catégorie(s) décrivent le mieux votre / vos domaine(s) de recherche dans le domaine de la douleur (sélectionnez toutes les réponses qui s'appliquent)?

- Fondamentale (p.e. recherche dans le domaine de la douleur sur, l'ADN, les cellules, les protéines, les molécules)
- Clinique (p.e. recherche dans le domaine de la douleur centrée sur les personnes atteintes de douleur – comme l'expérience vécue, évaluations, interventions, mesures)
- Translationnelle (p.e. recherche sur la douleur qui fait le lien entre recherche fondamentale et clinique)
- Translationnelle de clinique à la communauté élargie (p.e. recherche sur la douleur qui fait le lien entre recherche clinique et le grand public/les travailleurs en santé/décideurs politiques)

Q7: Quelle est votre source de financement principale pour soutenir votre salaire en tant qu'étudiant-chercheur (sélectionnez toutes les réponses applicables)?

- Bourse d'études supérieures (p.e. d'un des trois organismes - Instituts de Recherche en Santé du Canada, Conseil de recherches en sciences humaines, Conseil de recherches en sciences naturelles et en génie du Canada)
- Bourse d'études graduées provinciale (p.e. Bourse d'études supérieures de l'Ontario, Fonds de recherche en santé du Québec, etc.)
- Bourse de bienfaisance / sans but lucratif (p.e. Société Arthrite)
- Prix compétitif interne à un établissement universitaire
- Financement interne non compétitif de l'établissement universitaire (par exemple, financement du superviseur ou du département)
- Je ne reçois actuellement pas de financement
- Autre (Veuillez préciser) : [ouvrir la zone de texte]

Q8: Est-ce que votre propre expérience avec la douleur (p.e. étant vous-même une personne avec de la douleur, ou ayant un proche vivant avec de la douleur) vous a motivé à faire de la recherche dans le domaine de la douleur?

- Oui
- Non

Pour le reste du questionnaire, veuillez utiliser la définition suivante de l'engagement des patients, telle que décrite par les Instituts de Recherche en Santé du Canada et la Stratégie de recherche axée sur le patient. L'engagement des patients à la recherche implique une « collaboration significative et active à la gouvernance, à l'établissement de priorités, à la réalisation de la recherche et à l'application des connaissances. Selon le contexte, la recherche axée sur le patient peut également faire participer des gens qui sont porte-paroles de communautés touchées en particulier. » Veuillez passer à la prochaine question.

Q9: Selon votre perspective en tant qu'étudiant-chercheur, à quel point l'engagement des patients est important en recherche sur la douleur?

- pas du tout
- un peu
- modérément
- beaucoup
- extrêmement

Q10: Quelle formation avez-vous reçu sur l'engagement des patients en recherche (choisissez toutes les réponses qui s'appliquent)?

- Formation formelle faisant partie de votre programme académique (p.e. partie d'un cours académique)
- Formation formelle en dehors de votre programme académique (p.e. un atelier lors d'une conférence, un séminaire en ligne)
- Formation informelle en dehors de votre programme académique (p.e. mentorat de la part d'un de vos pairs/superviseur en dehors de votre programme académique)
- Autre (Veuillez décrire) : [ouvrir la zone de texte]
- Je n'ai pas reçu de formation sur l'engagement des patients en recherche

Q11: Est-ce que votre superviseur principal inclut l'engagement des patients dans sa recherche?

- Oui
- Non
- Je ne suis pas certain.e.

Q12: Dans quelle mesure connaissez-vous l'engagement des patients en recherche dans son ensemble?

- pas du tout
- un peu
- modérément
- beaucoup
- extrêmement

Q13: À quel point avez-vous confiance en vos capacités de mise en œuvre de mesures d'engagement des patients en recherche?

- pas du tout
- un peu
- modérément
- beaucoup
- extrêmement

Q14: À quelle fréquence avez-vous mis en œuvre des mesures d'engagement des patients en recherche dans des projets de recherche dans le domaine de la douleur où vous êtes l'investigateur principal (p.e. votre recherche doctorale)?

- jamais
- rarement
- des fois
- souvent
- toujours

Q15: À quelle fréquence avez-vous mis en œuvre des mesures d'engagement des patients en recherche dans des projets de recherche dans le domaine de la douleur où vous n'êtes pas l'investigateur principal (p.e. comme collaborateur sur un projet)?

- jamais
- rarement
- des fois
- souvent
- toujours

Q16: Comment avez-vous mis en œuvre des mesures d'engagement des patients en recherche, dans des projets où **vous êtes le chercheur principal** (p.e. votre recherche doctorale)? Choisissez toutes les réponses applicables :

- Impliquer un patient dans la phase de planification d'une recherche
- Impliquer un patient pour conseiller ou aider avec le recrutement de participants ou avec du matériel destiné aux participants (p.e. formulaire de consentement, matériel de recrutement, etc.)
- Impliquer un patient dans la collecte ou les analyses de données
- Impliquer un patient dans la transmission des connaissances (p.e. présentation en conférence)
- Impliquer un patient comme consultant
- Présenter des idées à des patients pour commentaires et rétroactions
- Impliquer un patient sur mon comité de thèse
- Impliquer un patient dans l'écriture d'une demande de bourse
- Autre (veuillez décrire) : [ouvrir la zone de texte]
- Je n'ai pas mis en œuvre de mesure d'engagement des patients en recherche dans un projet où je suis le chercheur principal

Q17: Comment avez-vous mis en œuvre des mesures d'engagement des patients en recherche, dans des projets où **vous n'êtes pas le chercheur principal** (p.e. comme collaborateur sur un projet)? Choisissez toutes les réponses applicables :

- Impliquer un patient dans la phase de planification d'une recherche
- Impliquer un patient pour conseiller ou aider avec le recrutement de participants ou avec du matériel destiné aux participants (p.e. formulaire de consentement, matériel de recrutement, etc.)
- Impliquer un patient dans la collecte ou les analyses de données
- Impliquer un patient dans la transmission des connaissances (p.e. présentation en conférence)
- Impliquer un patient comme consultant
- Présenter des idées à des patients pour commentaires et rétroactions
- Impliquer un patient sur mon comité de thèse
- Impliquer un patient dans l'écriture d'une demande de bourse
- Autre (veuillez décrire) : [ouvrir la zone de texte]
- Je n'ai pas mis en œuvre de mesure d'engagement des patients en recherche dans un projet où je ne suis pas le chercheur principal

Q18: Quels obstacles réels ou perçus avez-vous rencontrés lors de la mise en œuvre de mesures d'engagement des patients dans la recherche sur la douleur en tant qu'étudiant-chercheur (sélectionnez tout ce qui s'applique)?

- Manque de financement pour rembourser un patient partenaire ou lui fournir une compensation pour sa participation
- Incertitude quant à la valeur de l'engagement des patients

- Je ne sais pas comment mettre en œuvre concrètement l'engagement des patients
- Manque de confiance pour mettre en œuvre des mesures d'engagement des patients
- Je ne sais pas comment trouver des patients partenaires
- Manque de soutien du superviseur
- Manque de soutien de l'institution
- Je n'ai rencontré aucun obstacle
- Autre (veuillez décrire) : [ouvrir la zone de texte]

Q19: Quels facilitateurs réels ou perçus avez-vous rencontrés lors de la mise en œuvre de mesures d'engagement des patients dans la recherche sur la douleur en tant qu'étudiant-chercheur (sélectionnez tout ce qui s'applique)?

- Disponibilité de financement pour rembourser un patient partenaire ou lui fournir une compensation pour sa participation
- Réaliser la valeur de l'engagement des patients
- Connaissances sur comment mettre en œuvre concrètement des mesures d'engagement des patients
- Confiance pour mettre en œuvre des mesures d'engagement des patients
- Habilité à trouver des patients partenaires
- Soutien du superviseur
- Soutien de l'institution
- Je n'ai rencontré aucun facilitateur
- Autre (veuillez décrire) : [ouvrir la zone de texte]
- Non applicable - Je n'ai pas mis en œuvre de mesure d'engagement de patients à la recherche sur la douleur en tant qu'étudiant-chercheur

Q20: Quelles sont vos recommandations pour améliorer la mise en œuvre de l'engagement des patients en recherche parmi les étudiants-chercheurs conduisant des recherches dans le domaine de la douleur? [ouvrir la zone de texte]
