# Supplementary material for: The State of Patient Engagement among Pain Research Trainees in Canada: Results of a National Web-Based Survey
Source: Can J Pain. 2022 Oct 19;6(1):185–94. doi: 10.1080/24740527.2022.2115879 (PMC9586693; doi:10.1080/24740527.2022.2115879)
Supplement: Supplemental Material [file UCJP_A_2115879_SM1844.pdf]

Supplemental File 1 – Survey (English)

Q1: What is your age (in years)? [Open text box]

Q2: How do you self-identify in terms of gender?

- Woman
- Man
- Gender fluid, non-binary, and/or Two-Spirit
- I do not identify as any options mentioned above (please describe how you self-identify): [Open text box]

Q3: In what province/territory is your current academic institution located?

- Alberta
- British Colombia
- Manitoba
- New Brunswick
- Newfoundland and Labrador
- Northwest Territories
- Nova Scotia
- Nunavut
- Ontario
- Prince Edward Island
- Quebec
- Saskatchewan
- Yukon

Q4: Which category best describes your current status as a trainee?

- Undergraduate student, non-health profession
- Undergraduate student, health profession (e.g. RN, MD)
- Masters student, non-health profession
- Masters student, health profession (e.g. PT, OT)
- Combined health profession program and Masters student (e.g. MD/MSc)
- PhD student/candidate, non-health profession
- PhD student/candidate, health profession (e.g. Clinical Psychology)
- Combined health profession program and PhD student/candidate (e.g. MD/PhD)
- Post-doctoral fellow
- Resident (e.g. Resident Physician)
- Other (please describe): [Open text box]

Q5: What is your primary research supervisor’s stage of career?

- Early career (e.g. full time, independent research appointment for 0-5 years)
- Mid career (e.g. full time, independent research appointment for 5-15 years)
- Senior career (e.g. full time, independent research appointment for > 15 years)
- Not sure

Q6: What category/categories best describe your area(s) of pain research (select all that apply)?

- Basic (e.g. pain research that focuses on DNA, cells, proteins, or molecules)
- Clinical (e.g. pain research that focuses on individuals with pain – such as lived experience, assessment, interventions, or measurement)
- Basic to clinical translational (sometimes called ‘bench to bedside’) (e.g. pain research that bridges the gap between basic and clinical research)
- Clinical to the broader community translational (e.g. pain research that bridges the gap between clinical research and the general public/healthcare providers/policy makers)
- Other (please describe): [Open text box]

Q7: How do you currently receive funding to support your salary as a trainee (select all that apply)?

- Canada graduate scholarship (e.g. from a tri-council agency – Canadian Institutes of Health Research, Social Sciences and Humanities Research Council, National Sciences and Engineering Research Council)
- Provincial graduate scholarship (e.g. Ontario Graduate Scholarship, Fonds de la recherche en santé du Québec, etc.)
- Charity/Non-for-profit scholarship (e.g. Arthritis Society)
- Competitive internal academic institution award
- Non-competitive internal funding package from academic institution (e.g. funding from supervisor or department)
- Other (please describe): [Open text box]
- I do not currently receive funding

Q8: Has your own experience with pain (e.g. as a person with pain yourself or having a loved one or family member with pain) motivated you to do research in the field of pain?

- Yes
- No

For the remainder of this survey, please use the following definition of patient engagement, as described by the Canadian Institutes of Health Research and the Strategy for Patient Oriented Research. Patient engagement in research involves “[m]eaningful and active collaboration in governance, priority setting, conducting research and knowledge translation. Depending on the context, patient-oriented research may also engage people [as partners] who bring the collective voice of specific, affected communities.” Patient “is overarching and is inclusive of individuals with personal experience of a health issue and informal caregivers, including family and friends.” Please proceed to the next question.

Q9: How important is patient engagement in pain research from your perspective as a trainee?

- Not at all
- Slightly
- Moderately
- Very
- Extremely

Q10: What training have you received on patient engagement in research (select all that apply)?

- Formal training as part of your academic program (e.g. as part of an academic course)
- Formal training outside of your academic program (e.g. a conference workshop or webinar) Informal training as part of your academic program (e.g. mentorship from a peer/supervisor as part of your academic program)
- Informal training outside of your academic program (e.g. mentorship from a peer/non-supervisor outside of your academic program)
- Other (please describe): [Open text box]
- I have not received any training on patient engagement in research

Q11: Does your primary research supervisor implement patient engagement in their research?

- Yes
- No
- Not sure

Q12: How knowledgeable are you about patient engagement in research as a whole?

- Not at all
- Slightly
- Moderately
- Very
- Extremely

Q13: How confident are you in your ability to implement patient engagement in research?

- Not at all
- Slightly
- Moderately
- Very
- Extremely

Q14: How often have you implemented patient engagement in research on projects in the field of pain where you are the primary researcher (e.g. your thesis research)?

- Never
- Rarely
- Sometimes
- Often
- Always

Q15: How often have you implemented patient engagement in research on projects in the field of pain where you are not the primary researcher (e.g. as a collaborator on a project)?

- Never
- Rarely
- Sometimes
- Often
- Always

Q16: How have you implemented patient engagement in research on projects where you are the primary researcher (e.g. your thesis research)? Select all that apply:

- Involving a patient in the planning stage of research
- Involving a patient to advise or assist with participant recruitment or with materials for participants (e.g. informed consent forms, recruitment materials, etc.)
- Involving a patient in data collection or analysis
- Involving a patient in manuscript writing (e.g., as a co-author)
- Involving a patient in knowledge translation (e.g. presentation at a conference)
- Involving a patient on a consultant basis
- Presenting ideas to patients for input/feedback
- Involving a patient on my thesis committee
- Involving a patient in grant writing
- Other (please describe): [Open text box]
- I have not implemented patient engagement in research on a project when I am the primary researcher

Q17: How have you implemented patient engagement in research on projects where you are not the primary researcher (e.g. as a collaborator on a project)? Select all that apply:

- Involving a patient in the planning stage of research
- Involving a patient to advise or assist with participant recruitment or with materials for participants (e.g. informed consent forms, recruitment materials, etc.)
- Involving a patient in data collection or analysis
- Involving a patient in manuscript writing (e.g. as a co-author)
- Involving a patient in knowledge translation (e.g. presentation at a conference)
- Involving a patient on a consultant basis
- Presenting ideas to patients for input/feedback
- Involving a patient in grant writing
- Other (please describe): [Open text box]
- I have not implemented patient engagement in research on a project when I am not the primary researcher

Q18: What actual or perceived barriers have you encountered when implementing patient engagement in pain research as a trainee (select all that apply)?

- Lack of funding to reimburse a patient partner or provide them with compensation for participation
- Unsure about the value of patient engagement
- Unsure how to practically implement patient engagement
- Lack of confidence to implement patient engagement
- Don’t know how to find patient partners
- Lack of support from supervisor
- Lack of support from department
- Lack of support form institution
- I have not encountered any barriers
- Other (please describe): [Open text box]

Q19: What actual or perceived facilitators have you encountered when implementing patient engagement in pain research as a trainee (select all that apply)?

- Availability of funding to reimburse a patient partner or provide them with compensation for participation
- See the value of patient engagement
- Knowledge of how of how to practically implement patient engagement
- Confidence to implement patient engagement
- Ability to find patient partners
- Support from supervisor
- Support from department
- Support from institution
- I have not encountered any facilitators
- Other (please describe): [Open text box]
- Not applicable – I have not implemented patient engagement in pain research as a trainee

Q20: What are your recommendations to improve the implementation of patient engagement in research among trainees who are conducting research in the field of pain? [Open text box]
